# Find Duplicates among the PubMed, EMBASE, and Cochrane Library Databases in Systematic Review

**DOI:** 10.1371/journal.pone.0071838

**Published:** 2013-08-20

**Authors:** Xingshun Qi, Man Yang, Weirong Ren, Jia Jia, Juan Wang, Guohong Han, Daiming Fan

**Affiliations:** 1 Xijing Hospital of Digestive Diseases, Fourth Military Medical University, Xi’an, China; 2 Department of Gastroenterology, No. 463 Hospital of Chinese PLA, Shenyang, China; University of Illinois-Chicago, United States of America

## Abstract

**Background:**

Finding duplicates is an important phase of systematic review. However, no consensus regarding the methods to find duplicates has been provided. This study aims to describe a pragmatic strategy of combining auto- and hand-searching duplicates in systematic review and to evaluate the prevalence and characteristics of duplicates.

**Methods and Findings:**

Literatures regarding portal vein thrombosis (PVT) and Budd-Chiari syndrome (BCS) were searched by the PubMed, EMBASE, and Cochrane library databases. Duplicates included one index paper and one or more redundant papers. They were divided into type-I (duplicates among different databases) and type-II (duplicate publications in different journals/issues) duplicates. For type-I duplicates, reference items were further compared between index and redundant papers. Of 10936 papers regarding PVT, 2399 and 1307 were identified as auto- and hand-searched duplicates, respectively. The prevalence of auto- and hand-searched redundant papers was 11.0% (1201/10936) and 6.1% (665/10936), respectively. They included 3431 type-I and 275 type-II duplicates. Of 11403 papers regarding BCS, 3275 and 2064 were identified as auto- and hand-searched duplicates, respectively. The prevalence of auto- and hand-searched redundant papers was 14.4% (1640/11403) and 9.1% (1039/11403), respectively. They included 5053 type-I and 286 type-II duplicates. Most of type-I duplicates were identified by auto-searching method (69.5%, 2385/3431 in PVT literatures; 64.6%, 3263/5053 in BCS literatures). Nearly all type-II duplicates were identified by hand-searching method (94.9%, 261/275 in PVT literatures; 95.8%, 274/286 in BCS literatures). Compared with those identified by auto-searching method, type-I duplicates identified by hand-searching method had a significantly higher prevalence of wrong items (47/2385 versus 498/1046, p<0.0001 in PVT literatures; 30/3263 versus 778/1790, p<0.0001 in BCS literatures). Most of wrong items originated from EMBASE database.

**Conclusion:**

Given the inadequacy of a single strategy of auto-searching method, a combined strategy of auto- and hand-searching methods should be employed to find duplicates in systematic review.

## Introduction

Systematic review is characterized as explicitly formulated, reproducible, and up-to-date summary of the effects of health care interventions [1], [2]. It provides the top level of evidence for clinical decision [3], [4]. More than 2500 new systematic reviews every year can be retrieved in PubMed [5]. Compared with the traditional narrative review, the most prominent specialty of the systematic review is that literature search is comprehensive and literature selection is unbiased. Recently, the Preferred Reporting Items for Systematic reviews and Meta-Analyses (PRISMA) statement has recommended that a four-phase flow diagram should be employed for literature search and selection in systematic review [1]. The first phase is to identify all relevant literatures through databases and subsequently to remove the duplicates simultaneously recorded by different databases or published by different journals. The process of finding duplicates among databases is so critical that the researchers can avoid the repetitive evaluation of data from the same study and the readers can accurately understand the quantity of scientific publications in the field. Based on our previous systematic reviews [6], [7], [8], [9], a high prevalence of duplicates can be frequently observed among different databases. More importantly, not all duplicates can be readily found, because wrong information is occasionally recorded. However, no consensus regarding the methods to find duplicates and the prevalence of duplicates among different databases has been given yet.

Herein, we attempted to describe our methods to find duplicates among the PubMed, EMBASE, and Cochrane library databases in systematic review and to evaluate the prevalence and characteristics of duplicates.

## Methods

### Literature search

Literatures in two fields were retrieved to minimize the potential selection bias. They included “portal vein thrombosis” and “Budd-Chiari syndrome” literatures. The selection of the two fields was primarily attributed to our research interests in the two vascular disorders of the liver [6], [7], [8], [9], [10], [11]. QX searched the PubMed, EMBASE, and Cochrane library databases (from the database inception to November 12, 2012). Our search strategy aimed to maximize the quantity of literatures recorded by these databases. The search items were discussed by all review authors. For the literatures regarding portal vein thrombosis, the search items were: (portal vein thrombosis) OR (portal venous thrombosis) OR (portal vein obstruction) OR (portal venous obstruction). For the literatures regarding Budd-Chiari syndrome, the search items were: (budd chiari) OR (hepatic vein obstruction) OR (hepatic venous obstruction) OR (hepatic vein thrombosis) OR (hepatic venous thrombosis).

### Definitions and classifications of duplicates

Duplicates were divided into *type I* (duplicates among databases) and *II* (duplicate publications). *Type I* duplicates were defined as one paper was simultaneously recorded in one database twice or more times or in two or three databases (***see examples in*** ***Table 1***). *Type II* duplicates were defined as one study was published in different journals or issues. According to the type of publication, *type II* duplicates were classified as Abstract-Abstract, Abstract-Full text, and Full text-Full text. The first two types were often permitted, but the last one type was unethical in most of cases [12] (***see examples in*** ***Table 2***).

**Table 1:** Examples of type I duplicates (duplicates among databases).

Example	Index paper	Database	Redundant papers	Database	Acceptable causes	Unacceptable causes
No. 1	Ames PR. Recurrent abdominal thrombosis despite heparin thromboprophylaxis in a patient with transient eosinophilia. ***Clin Appl Thromb Hemost.*** 2011 ***Apr***;17(2):229–31.	PubMed	Ames PR***J***. Recurrent abdominal thrombosis despite heparin thromboprophylaxis in a patient with transient eosinophilia. ***Clinical and Applied Thrombosis/Hemostasis.*** 2011;17(2):229–31.	EMBASE	1) The author's middle name was missing in PubMed. 2) The journal's name was spelt in full style in EMBASE, but in abbreviated style in PubMed. 3) The publication date was expressed in “year” style in EMBASE, but in “year month” style in PubMed.	None
No. 2	Boylan JF, Klinck JR, Sandler AN, Arellano R, Greig PD, Nierenberg H, et al. Tranexamic acid reduces blood loss, transfusion requirements, and coagulation factor use in primary orthotopic liver transplantation. Anesthesiology. 1996 ***Nov;85***(5)***:1043–8; discussion 30A–31A.***	PubMed	Boylan JF, Klinck JR, Sandler AN, Arellano R, Greig PD, Nierenberg H, et al. Tranexamic acid reduces blood loss, transfusion requirements, and coagulation factor use in primary orthotopic liver transplantation. Anesthesiology ***[serial on the Internet]***. 1996; (5): ***Available from:*** http://onlinelibrary.wiley.com/o/cochrane/clcentral/articles/561/CN-00133561/frame.html **.** Boylan JF, Klinck JR, Sandler AN, Arellano R, Greig PD, Nierenberg H, et al. Tranexamic acid reduces blood loss, transfusion requirements, and coagulation factor use in primary orthotopic liver transplantation. Anesthesiology. 1996;***85***(5):***1043–8***.	Cochrane library (upper) EMBASE (lower)	1) The publication date was expressed in “year” style in EMBASE and Cochrane library, but in “year month” style in PubMed. 2) The page was expressed in different styles between PubMed and EMBASE.	1) The volume and page were missing in Cochrane library.
No. 3	***Goel P, Srivastava K, Das N, Bhatnagar V.*** The role of nitric oxide in portal hypertension caused by extrahepatic portal vein obstruction. ***J Indian Assoc Pediatr Surg.*** 2010 ***Oct;15***(4):***117–21***.	PubMed	***Bhatnagar V, Goel P, Srivastava K, Das N.*** The role of nitric oxide in portal hypertension caused by extrahepatic portal vein obstruction. ***Journal of Indian Association of Pediatric Surgeons [serial on the Internet].*** 2010; (4): ***Available from:*** http://onlinelibrary.wiley.com/o/cochrane/clcentral/articles/993/CN-00797993/frame.html **.**	Cochrane Library	1) The journal's name was spelt in full style in EMBASE, but in abbreviated style in PubMed. 2) The publication date was expressed in “year” style in EMBASE, but in “year month” style in PubMed.	1) The authors' order was wrong in Cochrane library. 2) The volume and page were missing in Cochrane library.
No. 4	Bjorkman JA, Ilebekk A, Jern C. Release of tissue-type plasminogen activator (t-PA) in the splanchnic circulation of the anaesthetised pig during high sympathetic tone. ***Thromb Res. 2010 Mar;125(3): e106–9.***	PubMed	Bjorkman JA, Ilebekk A, Jern C. Release of tissue-type plasminogen activator (t-PA) in the splanchnic circulation of the anaesthetised pig during high sympathetic tone. ***Thrombosis Research. 2009.***	EMBASE	1) The journal's name was spelt in full style in EMBASE, but in abbreviated style in PubMed.	1) The publication date was wrong in EMBASE. 2) The volume, issue, and page were missing in EMBASE.
No. 5	Cappellini MD, Grespi E, Cassinerio E, Bignamini D, Fiorelli G. Coagulation and splenectomy: ***a***n overview. ***Ann N Y Acad Sci.*** 2005;***1054***:317–24.	PubMed	Cappellini MD, Grespi E, Cassinerio E, Bignamini D, Fiorelli G. Coagulation and splenectomy: ***A***n overview. 2005. ***p***. 317–24.	EMBASE	1) The title was in uppercase in EMBASE, but in lowercase in PubMed. 2) The page was expressed in different styles.	1) The journal's name was missing in EMBASE. 2) The volume was missing in EMBASE.
No. 6	Choi JY, Lee JY, ***Lee JM, Kim SH, Lee MW, Han JK***, et al. Routine intraoperative Doppler sonography in the evaluation of complications after living-related donor liver transplantation. ***J Clin Ultrasound***. 2007 ***Nov-Dec***;35(9):483–90.	PubMed	Choi JY, Lee JY, ***Jeong ML, Se HK, Min WL, Joon KH,*** et al. Routine intraoperative Doppler sonography in the evaluation of complications after living-related donor liver transplantation. ***Journal of Clinical Ultrasound***. 2007;35(9):483–90.	EMBASE	1) The journal's name was spelt in full style in EMBASE, but in abbreviated style in PubMed. 2) The publication date was expressed in “year” style in EMBASE, but in “year month” style in PubMed.	1) The family and given names of authors were reversed in EMBASE.
No. 7	Akin O, Dixit D, Schwartz L. Bland and tumor thrombi in abdominal malignancies: ***m***agnetic resonance imaging assessment in a large oncologic patient population. ***Abdom Imaging.*** 2011 ***Feb***;36(1):62–8.	PubMed	***Engelbrecht M***, Akin O, Dixit D, Schwartz L. Bland and tumor thrombi in abdominal malignancies: ***M***agnetic resonance imaging assessment in a large oncologic patient population. ***Abdominal Imaging.*** 2011;36(1):62–8.	EMBASE	1) The title was in uppercase in EMBASE, but in lowercase in PubMed. 2) The journal's name was spelt in full style in EMBASE, but in abbreviated style in PubMed. 3) The publication date was expressed in “year” style in EMBASE, but in “year month” style in PubMed.	1) One author was wrongly added in EMBASE.
No. 8	***Bombeli T, Basic A, Fehr J***. Prevalence of hereditary thrombophilia in patients with thrombosis in different venous systems. ***Am J Hematol***. 2002 ***Jun***;70(2):126–32.	PubMed	***Gurney D, Lip GYH, Blann AD***. Prevalence of hereditary thrombophilia in patients with thrombosis in different venous systems. ***American Journal of Hematology***. 2002;70(2):126–32.	EMBASE	1) The journal's name was spelt in full style in EMBASE, but in abbreviated style in PubMed. 2) The publication date was expressed in “year” style in EMBASE, but in “year month” style in PubMed.	1) All authors were wrongly listed in EMBASE.
No. 9	Kimura K, Okuda K, Takara K, ***Matsutani S, Lesmana L***. Membranous obstruction of the portal vein. A case report. Gastroenterology. 1985 ***Feb***;88(2):571–***5***.	PubMed	Kimura K, Okuda K, Takara K. Membranous obstruction of the portal vein. A case report. Gastroenterology. 1985;88(2):571–***7***.	EMBASE	1) The publication date was expressed in “year” style in EMBASE, but in “year month” style in PubMed.	1) Two authors were missing in EMBASE. 2) The page was wrong in EMBASE.
No. 10	Ozbulbul NI, Yurdakul M, Tola M. ***Does the right inferior phrenic artery have a supplying role in liver c***irrhosis without hepatocellular carcinoma? A 64-slice CT study. ***Diagn Interv Radiol.*** 2011 ***Sep***;17(3):239–42.	PubMed	Ozbulbul NI, Yurdakul M, Tola M. ***C***irrhosis without hepatocellular carcinoma? A 64-slice CT study. ***Diagnostic and Interventional Radiology***. 2011;17(3):239–42.	EMBASE	1) The journal's name was spelt in full style in EMBASE, but in abbreviated style in PubMed. 2) The publication date was expressed in “year” style in EMBASE, but in “year month” style in PubMed.	1) Some words of the titles were missing in EMBASE.
No. 11	Senzol***o*** M, Cholongitas EC, Patch D, Burroughs AK. Update on the classification, assessment of prognosis and therapy of Budd-Chiari syndrome. ***Nat Clin Pract Gastroenterol Hepatol***. 2005 ***Apr***;2(4):182–90.	PubMed	Senzol***e*** M, Cholongitas EC, Patch D, Burroughs AK. Update on the classification, assessment of prognosis and therapy of Budd-Chiari syndrome. ***Nature Clinical Practice Gastroenterology and Hepatology***. 2005;2(4):182–90.	EMBASE	1) The journal's name was spelt in full style in EMBASE, but in abbreviated style in PubMed. 2) The publication date was expressed in “year” style in EMBASE, but in “year month” style in PubMed.	1) One author's name was wrongly spelt in EMBASE.

Notes:

– All examples originated from the literatures regarding portal vein thrombosis.

– In every example, the same study was simultaneously recorded by two or three databases.

– All literatures were expressed in Vancouver reference type.

– Bold and *italics* formatting indicated the different styles between index and redundant paper(s).

– In every example, the reference recorded by PubMed database had more complete information.

**Table 2:** Examples of type II duplicates (duplicate publications).

Example	References	Databases	No. Pts	Target population	Study objectives	Publication type
**No. 1**	Carr BI, Pancoska P, Branch RA. Tumor and liver determinants of prognosis in unresectable hepatocellular carcinoma: a ***large*** case cohort study. ***Hepatol Int. 2010;4(1):396–405*** *.*	PubMed	967	Unresectable and untransplantable, biopsy-proven hepatocellular carcinoma	Survival analysis	Full-text
	Carr BI, ***Buch SC, Kondragunta V*** *,* Pancoska P, Branch RA. Tumor and liver determinants of prognosis in unresectable hepatocellular carcinoma: a case cohort study. ***J Gastroenterol Hepatol. 2008 Aug;23(8 Pt 1):1259–66*** *.*	PubMed	967	Unresectable and untransplantable, biopsy-proven hepatocellular carcinoma	Survival analysis	Full-text
**No. 2**	Ban D, Shimada K, Nara S, Esaki M, Sakamoto Y, ***Kosuge T*** *.* Efficacy of hepatectomy and tumor thrombectomy for hepatocellular carcinoma with tumor thrombus extending to the main portal vein. ***Gastroenterology. 2009;136(5): A894.***	EMBASE	45	Tumor invasion of the first branch of the portal vein and tumor in the main portal trunk or the opposite-side portal branch	Efficacy of hepatectomy and tumor thrombectomy	Abstract
	Ban D, Shimada K, ***Yamamoto*** * Y*, Nara S, Esaki M, Sakamoto Y, et al. Efficacy of ***a*** hepatectomy and ***a*** tumor thrombectomy for hepatocellular carcinoma with tumor thrombus extending to the main portal vein. ***J Gastrointest Surg. 2009 Nov;13(11):1921–8***.	PubMed	45	Portal vein tumor thrombus extending to the first portal branch and main portal vein trunk	Efficacy of hepatectomy and tumor thrombectomy	Full-text
**No. 3**	Bartlett D, Lloyd C, Mirza D, McKiernan P, Newsome P. Nitisinone treatment reduces the need for iver transplantation in children with ***T***yrosinaemia ***T***ype 1 and is associated with improved post-transplant renal tubular function. ***Gut. 2010;59: A47–A8*** *.*	EMBASE	38	Tyrosinaemia Type 1	Efficacy of Nitisione	Abstract
	Bartlett D*C*, Lloyd C, Mirza D, McKiernan PJ, Newsome PN. Nitisinone treatment reduces the need for liver transplantation in children with ***t***yrosinaemia ***t***ype 1 and is associated with improved post-transplant renal tubular function. ***Hepatology. 2010;52:1030A–1A*** *.*	EMBASE	38	Tyrosinaemia Type 1	Efficacy of Nitisione	Abstract

Notes:

– All examples originated from the literature regarding portal vein thrombosis.

– In every example, the same study was published in two different journals.

– All literatures were expressed in Vancouver reference type.

– **Bold** and *italics* formatting indicated the different styles between index and redundant paper(s).

Duplicates consisted of one index paper and one or more redundant papers. For type I duplicates, index paper was considered as one paper of the duplicates had more accurate and/or adequate reference information; and for type II duplicates, index paper was considered as one paper of the duplicates was published earlier and/or had a larger sample size [13]. According to the number of redundant papers, duplicates were classified as follows: double duplicates were defined if only one redundant paper was found, triple duplicates if two redundant papers were found, quadruple duplicates if three redundant papers were found, and so on. According to the origin of index and redundant papers, duplicates were classified as PubMed-PubMed, PubMed-EMBASE, PubMed-Cochrane, EMBASE-EMBASE, EMBASE-Cochrane, Cochrane-Cochrane, and PubMed-EMBASE-Cochrane.

### Auto-search duplicates

QX imported all literatures retrieved by the three databases into an Endnote library (ENDNOTE X3, Thomson Reuters, USA). All literatures were expressed in Vancouver reference type. In the Endnote library, QX used the “*Find Duplicates*” command on the “*References*” menu to identify the auto-searched duplicates among the three databases. Prior to this step, “*Find Duplicates*” preferences could be defined on the “*Edit*” menu. To maximize the quantity of auto-searched duplicates, our preference was consistent with the Endnote default setting. In this setting, duplicates were identified as references of the same reference type with matching “author”, “title”, and “publication date” items, but “journal’s name”, “volume”, “issue”, and “page” items were not compared. QX further verified the accuracy of auto-searched duplicates.

### Hand-search duplicates

After auto-searched redundant papers were removed, the remaining literatures were alphabetically ordered according to the first authors’ names. Then, duplicates were identified among the literatures by the same first author. In details, if

Notably, if the first author’s name was wrongly spelt or missing or the authors’ order was reversed in some database, we would miss some duplicates. Accordingly, to minimize the quantity of missed duplicates, the literatures were also alphabetically ordered according to the titles. Then, duplicates were identified among the literatures with the same titles. YM and JJ were responsible for the literatures regarding portal vein thrombosis, and QX and RW for the literatures regarding Budd-Chiari syndrome. QX and YM were also responsible for rechecking the accuracy of their tasks. Disagreement would be resolved by discussion among the four review authors.

### Difference between index and redundant papers of type I duplicates

We just compared the difference of reference items between index and redundant papers of type I duplicates, but not type II duplicates. This behavior was primarily attributed to the fact that nearly all type II duplicates had different journal’s name, volume, issue, and page between index and redundant papers. QX and YM extracted the detailed information of type I duplicates (i.e., author, title, journal’s name, publication date, volume, issue, and page) into an Excel table (Microsoft Office Excel 2003, Microsoft Corporation, USA). Then, QX and YM compared the difference of reference items between index and redundant papers, and identified “acceptable or unacceptable” duplicate publications in order to distinguish whether or not they had wrong information.

Difference between index and redundant paper(s) would be considered acceptable to readers and reference reviewers, if the information was expressed in different styles. These different styles included: 1) punctuation, space, or case was different; 2) author’s middle name was omitted; 3) title of non-English language paper and non-English language journal’s name were translated into different words, but their meanings were identical; 4) journal’s name was expressed in full or abbreviated style; 5) publication date was expressed in “year” or “year month (day)” style; and 6) volume, issue, or page was expressed in different styles, but their meanings were identical (***see examples in*** ***Table 1***).

Difference between index and redundant paper(s) would be considered unacceptable to readers and reference reviewers, if the information was wrongly expressed. These wrong styles included: 1) author’s name and order, title of English language paper, and/or journal’s name was wrongly recorded, added, or missing; and 2) publication date, volume, issue, and/or page was wrong or missing (***see examples in*** ***Table 1***). QX further obtained the full-texts of the corresponding papers to identify the database which the wrong information originated from. In the cases where some full-text papers could not be obtained, we were uncertain about which database the wrong information originated from.

### Data analysis

The count data and/or percentage were reported in texts or bar charts. The prevalence of duplicates with 95% confidence intervals (CI) was calculated as follows:



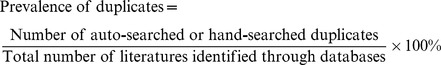


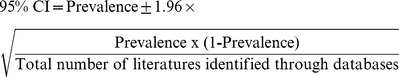



The proportion of type I and II duplicates was compared between auto-searching and hand-searching methods. The prevalence of different and wrong items in type I duplicates was compared between auto-searching and hand-searching methods. Two-tailed P values <0.05 were considered statistically significant. The statistical analyses were performed in SPSS 12.0 (SPSS Inc, Chicago, Ill).

## Results

### Portal vein thrombosis literatures

**Figure 1:**
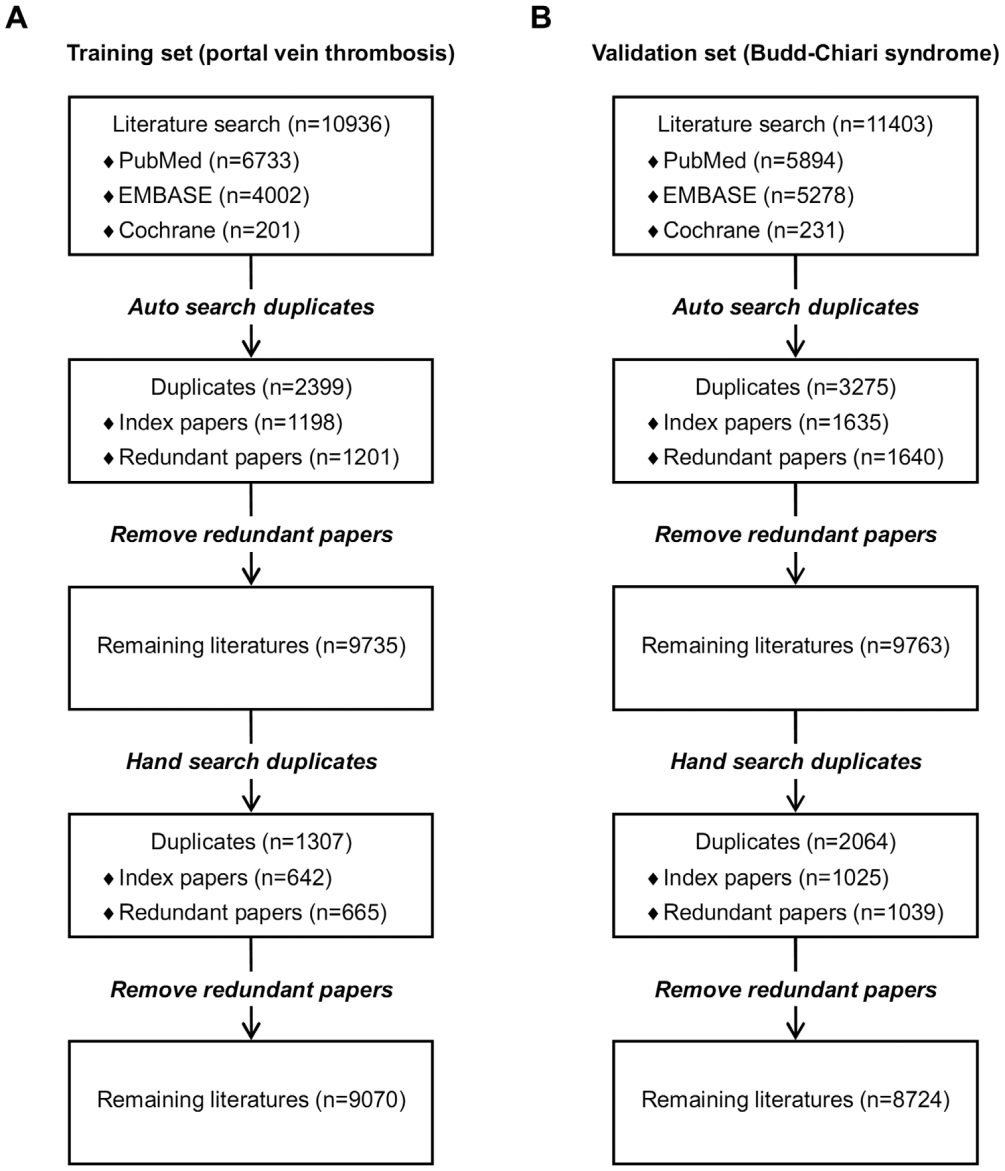
Study flowchart of finding duplicates in the literatures regarding portal vein thrombosis (panel A) and Budd-Chiari syndrome (panel B).

Overall, 10936 papers were identified via the three databases, including 6733 from PubMed database, 4002 from EMBASE database, and 201 from Cochrane library database (***Figure 1A***).

#### Auto-searched duplicates

Initially, 2401 papers were identified as auto-searched duplicates. Notably, 2 papers with the same author, title, and publication date were excluded from duplicates, because both of them reported different contexts in different issues. Thus, 2399 papers were auto-searched duplicates, including 1198 index papers and 1201 redundant papers (***Table 3***). The prevalence of auto-searched redundant papers was 11.0% (95%CI: 10.4%–11.6%).

**Table 3:** Characteristics of duplicates in literatures regarding portal vein thrombosis and Budd-Chiari syndrome.

Characteristics	Portal vein thrombosis			Budd-Chiari syndrome	
	Auto-searched duplicates	Hand-searched duplicates	Auto-searched duplicates		Hand-searched duplicates
No. total papers	2399	1307	3275		2064
– Index papers	1198	642	1635		1025
– Redundant papers	1201	665	1640		1039
*According to the type of duplicates*					
– Type I duplicates	2385	1046	3263		1790
– Type II duplicates	14	261	12		274
*According to the number of redundant papers*					
– Double duplicates (type I/II)	2392 (2378/14)	1242 (1022/220)	3262 (3250/12)		2022 (1772/250)
– Triple duplicates (type I/II)	3 (3/0)	57 (24/33)	9 (9/0)		42 (18/24)
– Quadruple duplicates (type I/II)	4 (4/0)	8 (0/8)	4 (4/0)		0 (0/0)
*According to the origin of duplicates*					
– PubMed-PubMed (type I/II)	4 (0/4)	28 (0/28)	2 (0/2)		24 (2/22)
– PubMed-EMBASE (type I/II)	2373 (2371/2)	939 (856/83)	3259 (3259/0)		1794 (1701/93)
– PubMed-Cochrane (type I/II)	0 (0/0)	113 (108/5)	0 (0/0)		42 (42/0)
– EMBASE-EMBASE (type I/II)	20 (12/8)	180 (35/145)	12 (2/10)		161 (2/159)
– EMBASE-Cochrane (type I/II)	0 (0/0)	32 (32/0)	0 (0/0)		28 (28/0)
– Cochrane-Cochrane (type I/II)	2 (2/0)	0 (0/0)	2 (2/0)		0 (0/0)
– PubMed-EMBASE-Cochrane (type I/II)	0 (0/0)	15 (15/0)	0 (0/0)		15 (15/0)
*According to the publication type of type II duplicates*					
– Abstract-Abstract	8	64	8		80
– Abstract-Full text	0	137	0		127
– Full text-Full text	6	60	4		67

Notes: Type I duplicates represent duplicates among databases; type II duplicates represent duplicate publications in different journals/issues.

Of 2385 type I duplicates, 14 had the completely same items between index and redundant papers. The remaining 2371 duplicates had at least one different item between index and redundant papers. Publication date (92.8%, 2213/2385) was the most commonly different item, followed by journal’s name (88.7%, 2115/2385), title (31.2%, 744/2385), page (5.8%, 139/2385), issue (3.0%, 71/2385), volume (0.9%, 21/2385), and author (0.6%, 14/2385) (***Table 4***). Only 2.0% (47/2385) of duplicates were considered unacceptable due to wrong information. Page (1.8%, 43/2385) was the most commonly wrong item, followed by issue (1.0%, 23/2385), volume (0.9%, 21/2385), publication date (0%, 0/2385), journal’s name (0%, 0/2385), title (0%, 0/2385), and author (0%, 0/2385).

**Table 4:** Type I duplicates – difference between index and redundant papers.

Items	Portal vein thrombosis			Budd-Chiari syndrome	
	Auto-searched duplicates	Hand-searched duplicates	Auto-searched duplicates		Hand-searched duplicates
No. type I duplicates	2385	1046	3263		1790
*Author item*					
– Same	2371	491	3235		788
– Different (Acceptable/Unacceptable)	14 (14/0)	555 (269/286)	28 (28/0)		1002 (358/644)
*Title item*					
– Same	1641	500	2252		728
– Different (Acceptable/Unacceptable)	744 (744/0)	546 (517/29)	1011 (1011/0)		1062 (1025/37)
*Journal's name item*					
– Same	270	137	416		267
– Different (Acceptable/Unacceptable)	2115 (2115/0)	909 (905/4)	2847 (2847/0)		1523 (1519/4)
*Publication date item*					
– Same	172	189	172		805
– Different (Acceptable/Unacceptable)	2213 (2213/0)	857 (841/16)	3091 (3091/0)		985 (977/8)
*Volume item*					
– Same	2364	885	3243		1693
– Different (Acceptable/Unacceptable)	21 (0/21)	161 (0/161)	20 (4/16)		97 (5/92)
*Issue item*					
– Same	2314	1012	3130		1718
– Different (Acceptable/Unacceptable)	71 (48/23)	34 (12/22)	133 (115/18)		72 (30/42)
*Page item*					
– Same	2246	815	3109		1630
– Different (Acceptable/Unacceptable)	139 (96/43)	231 (46/185)	154 (124/30)		160 (86/74)

Notes: Type I duplicates represent duplicates among databases.

**Figure 2:**
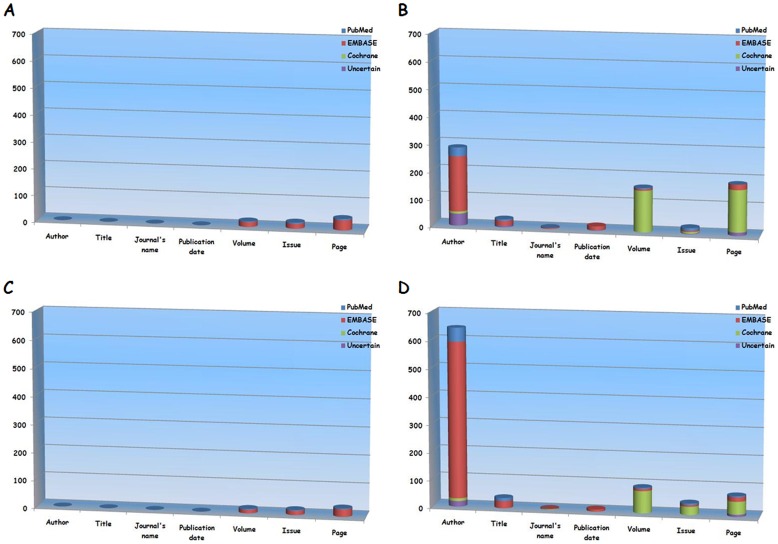
Proportion of wrong information of auto-searched (panel A) and hand-searched (panel B) type I duplicates from the literatures regarding portal vein thrombosis and that of auto-searched (panel C) and hand-searched (panel D) type I duplicates from literatures regarding Budd-Chiari syndrome.

EMBASE database had the highest proportion of wrong information regarding page, issue, and volume items (***Figure 2A***).

#### Hand-searched duplicates

After auto-searched redundant papers were removed, 1307 papers were further identified as hand-searched duplicates, including 642 index papers and 665 redundant papers (***Table 3***). The prevalence of hand-searched redundant papers was 6.1% (95%CI: 5.6%–6.5%).

Of 1046 type I duplicates, all had at least one different item between index and redundant papers. Journal’s name (87.1%, 909/1046) was the most commonly different item in hand-searched duplicates, followed by publication date (82.1%, 857/1046), author (53.2%, 555/1046), title (52.3%, 546/1046), page (22.1%, 231/1046), volume (15.4%, 161/1046), and issue (3.3%, 34/1046) (***Table 4***). 47.6% (498/1046) of duplicates were considered unacceptable due to wrong information. Author (27.4%, 286/1046) was the most commonly wrong item in hand-searched duplicates, followed by page (17.7%, 185/1046), volume (15.4%, 161/1046), title (2.8%, 29/1046), issue (2.1%, 22/1046), publication date (1.5%, 16/1046), and journal’s name (0.4%, 4/1046).

EMBASE database had the highest proportion of wrong information regarding author, title, journal, and publication date items. Cochrane library database had the highest proportion of wrong information regarding volume and page items. PubMed database had the highest proportion of wrong information regarding issue item (***Figure 2B***).

#### Comparison

The number of duplicates identified by auto-searching methods was larger than that identified by hand-searched duplicates (2399 versus 1307). Most of type I duplicates were identified by auto-searching methods (69.5%, 2385/3431). The proportion of type I duplicates among the auto-searched duplicates was significantly higher than that among the hand-searched duplicates (2385/2399 versus 1046/1307, p<0.0001). Nearly all type II duplicates were identified by hand-searching methods (94.9%, 261/275). The proportion of type II duplicates among the auto-searched duplicates was significantly lower than that among the hand-searched duplicates (14/2399 versus 261/1307, p<0.0001).

Compared with those identified by auto-searching methods, type I duplicates identified by hand-searching methods had a significantly higher prevalence of different and wrong items (different items: 2371/2385 versus 1046/1046, p = 0.008; wrong items: 47/2385 versus 498/1046, p<0.0001).

### Budd-Chiari syndrome literatures

Overall, 11403 papers were identified via the three databases, including 5894 from PubMed database, 5278 from EMBASE database, and 231 from Cochrane library database (***Figure 1B***).

#### Auto-searched duplicates

3275 papers were identified and verified as auto-searched duplicates, including 1635 index papers and 1640 redundant papers (***Table 3***). The prevalence of auto-searched redundant papers was 14.4% (95%CI: 13.7%–15.0%).

Of 3263 type I duplicates, 18 had the completely same items between index and redundant papers. The remaining 3245 duplicates had at least one different item between index and redundant papers. Publication date (94.7%, 3091/3263) was the most commonly different item, followed by journal’s name (87.3%, 2847/3263), title (31.0%, 1011/3263), page (4.7%, 154/3263), issue (4.1%, 133/3263), author (0.9%, 28/3263), and volume (0.6%, 20/3263) (***Table 2***). Only 0.9% (30/3263) of duplicates were considered unacceptable due to wrong information. Page (0.9%, 30/3263) was the most commonly wrong item, followed by issue (0.6%, 18/3263), volume (0.5%, 16/3263), publication date (0%, 0/3263), journal’s name (0%, 0/3263), title (0%, 0/3263), and author (0%, 0/3263).

EMBASE database had the highest proportion of wrong information regarding page, issue, and volume items (***Figure 2C***).

#### Hand-searched duplicates

After 1640 auto-searched redundant papers were removed, 2064 papers were further identified as hand-searched duplicates, including 1025 index papers and 1039 redundant papers (***Table 3***). The prevalence of hand-searched redundant papers was 9.1% (95%CI: 8.6%–9.6%).

Of 1790 type I duplicates, all had at least one different item between index and redundant papers. Journal’s name (85.1%, 1523/1790) was the most commonly different item in hand-searched duplicates, followed by title (59.3%, 1062/1790), author (56.0%, 1002/1790), publication date (55.0%, 985/1790), page (8.9%, 160/1790), volume (5.4%, 97/1790), and issue (4.0%, 72/1790) (***Table 4***). 43.5% (778/1790) of duplicates were considered unacceptable due to wrong information. Author (36.0%, 644/1790) was the most commonly wrong item in hand-searched duplicates, followed by volume (5.1%, 92/1790), page (4.1%, 74/1790), issue (2.3%, 42/1790), title (2.1%, 37/1790), publication date (0.4%, 8/1790), and journal’s name (0.2%, 4/1790).

EMBASE database had the highest proportion of wrong information regarding author, title, journal’s name, and publication date items. Cochrane library database had the highest proportion of wrong information regarding volume, issue, and page items (***Figure 2D***).

#### Comparison

The prevalence of duplicates identified by auto-searching methods was significantly higher than that identified by hand-searching methods (3275/11403 versus 2064/11403, p<0.0001). Most of type I duplicates were identified by auto-searching methods (64.6%, 3263/5053). The proportion of type I duplicates among the auto-searched duplicates was significantly higher than that among the hand-searched duplicates (3263/3275 versus 1790/2064, p<0.0001). Nearly all type II duplicates were identified by hand-searching methods (95.8%, 274/286). The proportion of type II duplicates among the auto-searched duplicates were significantly lower than that among the hand-searched duplicates (12/3275 versus 274/2064, p<0.0001).

Compared with those identified by auto-searching methods, type I duplicates identified by hand-searching methods had a significantly higher prevalence of different and wrong items (different items: 3245/3263 versus 1790/1790, p = 0.001; wrong items: 30/3263 versus 778/1790, p<0.0001).

## Discussion

**Figure 3:**
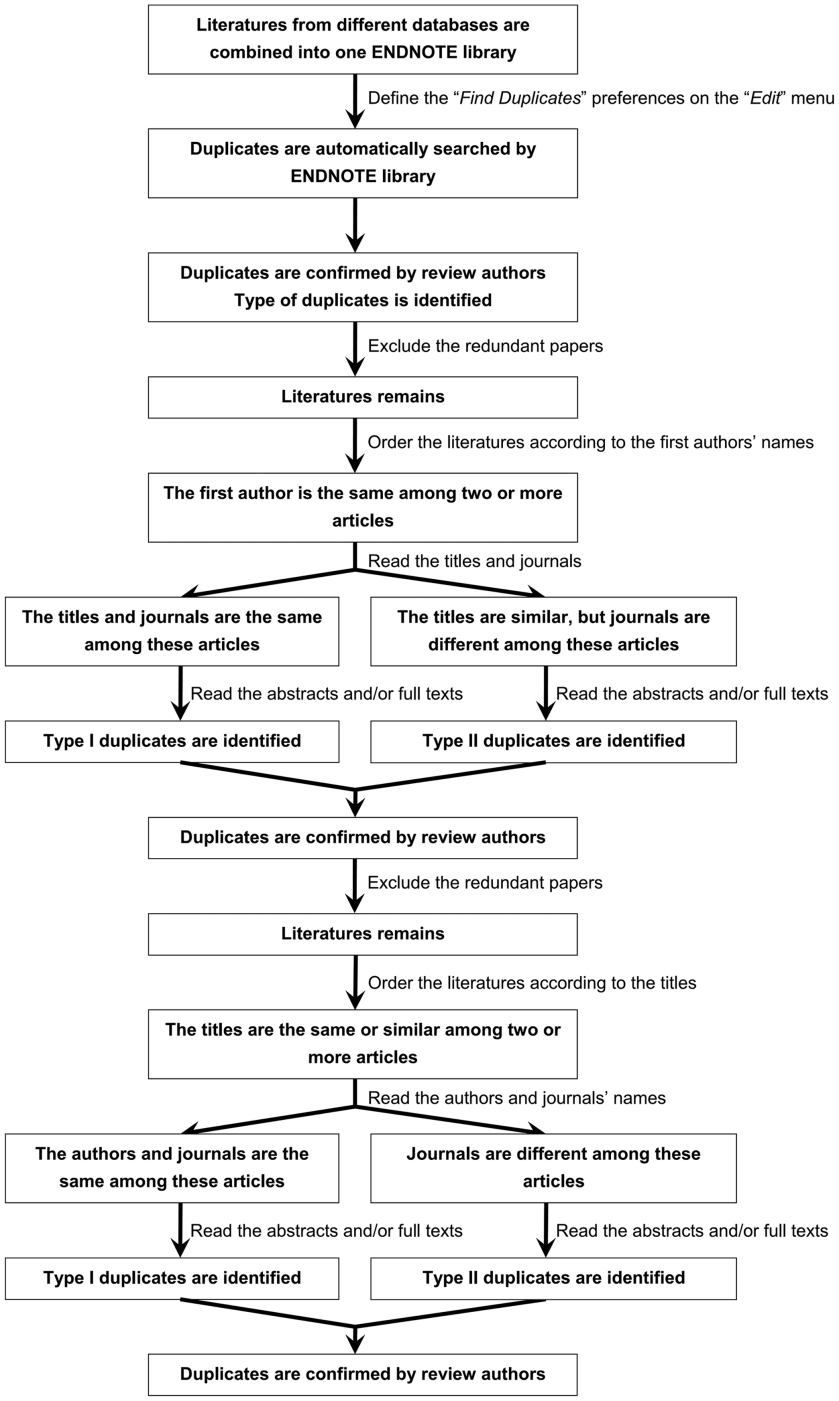
Simplified scheme to identify duplicates in systematic review. The scheme includes the third main steps. First, all literatures retrieved from different databases are combined into one Endnote library. In this Endnote library, “*Find Duplicates*” preferences are defined on “*Edit*” menu. Thus, duplicates can be automatically searched by Endnote library. Subsequently, the review authors should check the accuracy and identify the type of duplicates. Finally, the redundant papers are excluded. Considering that a single strategy of auto-searching method was inadequate, additional search should be very necessary. Second, the remaining literatures are alphabetically ordered according to the first authors’ names in the Endnote library. If the first authors were the same between two or more articles, the review authors would further read the titles, journals’ names, volumes, issues, and pages. Subsequently, if these articles had the same titles, journals’ names, and issues, they would be attributed to the type I duplicates. Notably, the review authors should identify whether the difference between index and redundant papers was acceptable or not. On the other hand, if these had the same or similar titles but different journals or issues, the review authors would further read the abstracts and/or full-texts to judge whether or not they could be attributed to the type II duplicates. Third, the remaining literatures were also alphabetically ordered according to the titles in the Endnote library. If the titles were the same between two or more articles, the review authors would further read the journals’ names, volumes, issues, and pages. Subsequently, if these articles had the same journals’ names and issues, they would be attributed to type I duplicates. Notably, the review authors should identify whether the difference between index and redundant papers was acceptable or not. On the other hand, if these articles had the same or similar titles but different journals or issues, the review authors would further read the abstracts and/or full-texts to judge whether or not they could be attributed to the type II duplicates. Finally, review authors should check the accuracy.

Finding duplicates among different databases is an indispensable and important phase of systematic review. The phase is not as easy as we expected according to our previous experiences of systematic reviews [6], [7], [8], [9]. However, little attention has been paid to this phase. To our knowledge, this study is the first systematic analysis of duplicates among the three databases commonly used by systematic review (i.e., PubMed, EMBASE, and Cochrane library database). We attempted to devise a scheme to identify duplicates in a systematic review (***Figure 3***). In this scheme, we employed two methods to find duplicates (i.e., auto-search and hand-search duplicates) and two approaches to find hand-searched duplicates (i.e., alphabetical order of literatures according to the first authors and titles). Indeed, the process of auto-searching duplicates can be easily accomplished by Endnote library software. By comparison, the process of hand-searching duplicates is really a time-consuming and careful work. Four review authors spent more than four weeks on finding hand-searched duplicates, and two of them also paid another two weeks for checking the accuracy of these works. Certainly, further studies should be designed to assess the practical utility of this method in systematic review.

A major finding of our study was that a large number of duplicates could be found among the three databases in systematic review. Notably, about 10% of literatures remained duplicates among the three databases after auto-searching duplicates, which strongly suggested the necessity of hand-searching duplicates in systematic review.

We further compared the difference of reference items between index and redundant papers of type I duplicates. Nearly all type I duplicates had different items between index and redundant papers. Regardless of the literatures regarding portal vein thromobosis or Budd-Chiari syndrome and auto-searched or hand-searched duplicates, “journal’s name”, “publication date”, and “title” were three most commonly different items. Most of them were acceptable, for example, journal’s name was expressed in full or abbreviated style, publication date was expressed in “year” or “year month” style, and titles used different punctuations and/or cases in different databases. This finding could be potentially explained by the fact that each database had its own special reference type. Other items were uncommon, but were mostly unacceptable. For example, author, volume, issue, or page was wrong or missing. These mistakes should be corrected, thereby decreasing the prevalence of type I duplicates.

In addition, our study explored the origin of wrong information in type I duplicates. Regardless of the literatures regarding portal vein thrombosis or Budd-Chiari syndrome, EMBASE database had the highest proportion of wrong information regarding author, title, journal, and publication date items. These mistakes in EMBASE database were severe (***see examples in*** ***Table 1***), because they not only misled the readers but also disrespected the researchers. Cochrane library database had the highest proportion of wrong information regarding volume and page items in type I duplicates. This was primarily due to the reference type of Cochrane library database (volume and page were not provided). By comparison, only a minority of wrong information in type I duplicates originated from PubMed database. These findings suggested the following: 1) the accuracy of reference information recorded by EMBASE database should be substantially improved; and 2) the same reference type among these databases may be beneficial for literature screening.

Auto-searching methods could identify a larger number of duplicates, especially type I duplicates. However, only a very small proportion of type II duplicates could be identified by auto-searching methods (5.1% in portal vein thrombosis literatures; and 4.8% in Budd-Chiari syndrome literatures). This phenomenon could be readily explained by the fact that the authors, titles, and publication years were often different between index and redundant papers among type II duplicates. Additionally, the wrong reference items were rarely observed among type I duplicates identified by auto-searching methods, but very frequently among those identified by hand-searching methods. This finding also suggested the limitation of auto-searching duplicates, in which “author”, “title”, and “publication date” items should be exactly matched between two literatures. Accordingly, the necessity of combining auto- and hand-searching methods should be fully recognized in finding duplicates in systematic reviews.

### Limitations

Several limitations of this study should be clearly recognized. First, the selection of portal vein thrombosis and Budd-Chiari syndrome literatures was based on our subjectivity. Accordingly, the conclusions achieved by analyzing these literatures might be unsuitable for the literatures from other fields. But it should be noted that we employed a comprehensive search strategy and literatures of two fields to strengthen our conclusions. And given that the results were similar between portal vein thrombosis and Budd-Chiari syndrome literatures, it was possible that these findings of our study might be generalizable. Certainly, further studies should be warranted to compare the frequency of wrong information from a random sample of literatures among the three databases. Second, only three databases were searched in our study. This behavior might underestimate the prevalence of duplicates among databases. However, given that PubMed, EMBASE, and Cochrane library were three most common databases used for systematic review, our results should be a representative sample. Third, only two approaches were employed in this study to identify hand-searched duplicates. It was not easy to find duplicates as both the first author’s name and title were different between index and redundant papers. Thus, the prevalence of duplicates might be underestimated. Fourth, a minority of full texts could not be obtained to identify the origin of wrong information. However, it should be noted that we tried our best to contact with the authors and seek help from our and other University libraries. And these unavailable full texts did not substantially influence our judgment on the proportion of wrong information in different databases.

## Conclusions

In conclusions, a high prevalence of duplicates could be identified among the PubMed, EMBASE, and Cochrane Library databases in systematic review. These findings were primarily attributed to the effect of a pragmatic strategy of combining auto- and hand-searching methods to find duplicates. Indeed, a single strategy of auto-searching method was inadequate to find duplicates, especially type II duplicates. In general, to enhance the transparency of systematic review, PRISMA might require the reporting of the detailed information regarding the methods to find duplicates and the quantity of duplicates identified by different methods. In addition, considering that wrong reference items were frequently observed in type I duplicates identified by hand-searching methods, we strongly recommended that the information of every reference should be strictly examined and carefully inputted by database administrators.

## Supporting Information

File S1 **Duplicates identified by auto-searching method in portal vein thrombosis literatures.**
(XLS

File S2 **Type-I duplicates identified by hand-searching method in portal vein thrombosis literatures.**
(XLS)

File S3 **Type-II duplicates identified by hand-searching method in portal vein thrombosis literatures.**
(XLS)

File S4 **Duplicates identified by auto-searching method in Budd-Chiari syndrome literatures.**
(XLS)

File S5 **Type-I duplicates identified by hand-searching method in Budd-Chiari syndrome literatures.**
(XLS)

File S6 **Type-II duplicates identified by hand-searching method in Budd-Chiari syndrome literatures.**
(XLS)
